# Preoperative NRI outperforms other time points in predicting prognosis of ESCC with neoadjuvant therapy

**DOI:** 10.3389/fnut.2025.1613868

**Published:** 2025-06-25

**Authors:** Xue Tang, Mei Yang

**Affiliations:** Department of Thoracic Surgery, West China Hospital, Sichuan University, Chengdu, Sichuan, China

**Keywords:** esophageal cancer, nutritional risk index, postoperative complication, prognosis, neoadjuvant therapy

## Abstract

**Background:**

Early studies reported that the nutritional risk index (NRI) is a prognostic factor in patients with various malignant tumors. Our study aims to demonstrate the prognostic role of the NRI by assessing the longitudinal clinical data of patients with esophageal squamous cell carcinoma (ESCC) who had undergone neoadjuvant therapy followed by esophagectomy.

**Materials and methods:**

Our study retrospectively investigated 319 ESCC patients who had been treated with neoadjuvant therapy before esophagectomy at West China Hospital, Sichuan University, between August 2016 and August 2021. The NRI was calculated based on the height, weight, and albumin levels of ESCC patients at three time points during the entire treatment course: before treatment, before esophagectomy, and post-esophagectomy.

**Results:**

A total of 319 patients with ESCC were included in the study. Logistic regression showed that ESCC patients with a low preoperative NRI had a higher postoperative complication rate than those with a high preoperative NRI (odds ratio [OR] = 2.324, 1.318–4.095, *p* = 0.004). The timing of malnutrition that affected survival was the preoperative NRI score. According to the multivariate analysis results, the preoperative NRI, rather than the pretreatment NRI or postoperative NRI, was an independent prognostic factor for overall survival (OS) (hazard ratio [HR] = 2.005, 1.070–3.760, *p* = 0.030) and disease-free survival (DFS) (HR = 1.736, 1.086–2.775, *p* = 0.021) in patients with ESCC.

**Conclusion:**

In patients with ESCC who underwent neoadjuvant therapy followed by esophagectomy, preoperative NRI might predict postoperative complications and survival outcomes of patients. Further clinical investigations are needed to determine the prognostic value of the NRI.

## 1 Introduction

Esophageal cancer (EC) is one of the most invasive malignant tumors and is the sixth leading cause of cancer-related death worldwide (1). The main pathological types of EC are esophageal squamous cell carcinoma (ESCC) and esophageal adenocarcinoma (EAC) (2). The incidence of EC has increased globally during recent years, and the overall five-year survival rate of EC patients is lower than 30% (3). Traditionally, the primary treatment for EC is esophagectomy. However, the early symptoms of EC are often easy to be neglected, resulting in many patients being diagnosed at an advanced local stage (4). Before esophagectomy, neoadjuvant therapy can shrink the primary tumor and improve the likelihood of a successful surgery, ultimately prolonging the survival of patients with EC; thus, neoadjuvant therapy combined with esophagectomy now becomes the mainstay of those patients with locally advanced EC (5). Neoadjuvant therapy has become a standard therapy for operable patients; however, maintaining proper nutrition throughout the treatment process can be complicated by various risk factors. These factors include dyscrasia, tract obstruction, neoadjuvant therapy-induced gastrointestinal adverse events, dysfunction of digestive organs after esophagectomy, and the invasive characteristic of EC. As a result, treating EC still presents severe challenges (6).

Assessing and providing nutrition are necessary steps in the treatment of malignancies (7). Various risk factors such as dyscrasia and obstruction induced by radiotherapy can make nutrition support hard in EC patients (8). In recent years, studies showed that in tumor treating, nutritional risk index (NRI) was a prognostic indicator in many malignancies, and the NRI could be easily calculated through the height, weight, and blood albumin levels of patients (9, 10). However, previous studies have not proven the prognostic value of NRI in ESCC patients who underwent neoadjuvant therapy followed by esophagectomy, and early studies have neglected to examine when the time point assessment of NRI is the most relevant to the postoperative complications and survival outcomes of ESCC patients. Thus, the current study attempted to prove the prognostic value of NRI in ESCC patients and to elucidate that NRI could not only be used in nutritional status evaluation but might also be helpful in facilitating more effective strategies and adjusting therapy in time.

## 2 Patients and methods

### 2.1 Study patients

Patients with histologically confirmed EC after surgical resection between August 2016 and August 2021 were included in this retrospective analysis at the West China Hospital, Sichuan University. The inclusion criteria were as follows: (1) patients were pathologically diagnosed with ESCC; (2) patients who had undergone esophagectomy; (3) patients who had been treated with neoadjuvant therapy (including neoadjuvant chemoradiotherapy or neoadjuvant chemotherapy) before esophagectomy; and (4) patients who had been followed up for a minimum of 6 months or until death. The exclusion criteria were as follows: (1) patients with cardio-gastroesophageal junction tumors, which are commonly recognized as gastric cancer; (2) non-R0 resected patients; and (3) patients with M1 stage verified during surgery. Finally, a total of 319 patients were included in this study. Patients included in the study were followed up until death or August 2021. This study was conducted in compliance with the National Health Care rules, with approval from the West China Hospital Ethics Committee (No. 2019632), and all patients provided their informed consent.

The medical, pathological, and clinical records of each ESCC patient were collected retrospectively from a maintained database, and the Union for International Cancer Control TNM Classification of Malignant Tumors (8th edition) and tumor regression grade (TRG) based on the Becker system were used for pathological diagnosis and disease classification (11, 12).

### 2.2 Nature of operation and extent of lymphadenectomy

All patients underwent McKeown esophagectomy according to the institutional protocol to ensure adequate proximal margins and conventional 2-field (abdominal and thoracic) lymph node excision. In this study, three-field lymph node dissection was not a common practice, and cervical lymph node dissection was selected for patients with suspected cervical lymph node metastases, as determined by preoperative CT and ultrasound.

### 2.3 Neoadjuvant therapy regimens

Neoadjuvant therapy was administered to the patients in accordance with national recommendations. Neoadjuvant chemotherapy involves 2 cycles of chemotherapy, with a 3-week break between each cycle. All patients received paclitaxel (175 mg/m^2^ body surface area, D1) and cisplatin (75 mg/m^2^ body surface area, D1) intravenously over 2 cycles.

As the aspect of the neoadjuvant chemoradiotherapy regimen, all patients received a total radiation dosage of 40–50.4 Gy in 23–28 fractions (1.8–2.0 Gy/fraction) and 2 cycles of the simultaneous chemotherapy drugs paclitaxel (175 mg/m2 body-surface area, D1, q3w) and cisplatin (75 mg/m^2^ body-surface area, D1, q3w). Intensity-modulated radiotherapy was used to provide radiation to all the patients. After neoadjuvant treatment, all patients underwent radical esophagectomy with two- or three-field lymphadenectomy using a minimally invasive or open method, followed by conduit reconstruction via the stomach.

### 2.4 NRI calculation

The exact NRI of each ESCC patient was calculated through the height, weight, and blood albumin as follows: (13).


$$\mathrm{NRI}=1.519\times \text{Albu}\min\;(g/L)+41.7\times \frac{\text{Current Weight}\;(\mathrm{kg})}{\text{Usual Weight}\;(\mathrm{kg})}$$

The NRI of each ESCC patient was collected at pretreatment, preoperatively, and postoperatively (within 1 month after esophagectomy) at three time points. For descriptive statistics, patients with ESCC were categorized into the no malnourishment risk group (NRI > 97.5) and malnourishment risk group (NRI ≤ 97.5), respectively (14–20). Furthermore, the BMI at different time points was also calculated based on Asian criteria, and patients with a BMI < 18.5 kg/m^2^ were considered as underweight (21).

### 2.5 Statistical analysis

Statistical Package for the Social Sciences (SPSS) version 26.0 (SPSS Inc., Chicago, IL, United States) was used for statistical analyses. The association of NRI with clinical or pathologic characteristics was calculated using Chi-squared or Fisher’s exact tests. Overall survival (OS) and disease-free survival (DFS) were defined as the duration from primary surgery to death or tumor recurrence. Survival curves were constructed using the Kaplan–Meier method, and the log-rank test was used to assess the differences between the groups. Logistic regression analysis was used to investigate risk factors for postoperative complications. The Cox proportional hazards model was used to conduct a multivariate analysis to confirm the independent prognostic factors of patients with ESCC. The odds ratio (OR), hazard ratio (HR), and 95% confidence interval (CI) were calculated, and a *p*-value <0.05 indicated statistical significance.

## 3 Results

### 3.1 Clinical characteristics of patients

A total of 319 patients who met the inclusion criteria were included in the study. Among all ESCC patients, 257 were male and 62 were female, with a mean age of 62.28 (range 44–80). Tumors were found in the upper thoracic esophagus in 48 patients, the middle thoracic esophagus in 191 patients, and the lower thoracic esophagus in 80 patients. Above all, the ESCC patients, the pathologic tumor T0,1 was found in 170 patients, and T2,3,4 in 149 patients. In another group of pathological tumor N stages, N-negative and N-positive were found in 200 and 119 ESCC patients, respectively. Based on the reported cutoff value of NRI, 97.5 was set as the cutoff value (14). Details of the patients’ clinical characteristics are shown in Table 1.

**Table 1:** Patient characteristics and NRI at different time points.

Factors	Cases	Pretreatment-NRI		*p*-value	Cases	Preoperative-NRI		*p*-value	Cases	Postoperative-NRI		*p*-value
	(*N* = 319)	High [*N* = 270]	Low [*N* = 49]		(*N* = 319)	High [*N* = 255]	Low [*N* = 64]		(*N* = 319)	High [*N* = 142]	Low [*N* = 177]	
Sex												
Female	62	53 (19.6%)	9 (18.4%)	0.837	62	54 (21.2%)	8 (12.5%)	0.117	62	33 (23.2%)	29 (16.4%)	0.124
Male	257	217 (80.4%)	40 (81.6%)		257	201 (78.8%)	56 (87.5%)		257	109 (76.8%)	148 (83.6%)	
Age												
>60	122	107 (39.6%)	15 (30.6%)	0.232	122	101 (39.6%)	21 (32.8%)	0.317	122	60 (42.3%)	62 (35.0%)	0.187
≤60	197	163 (60.4%)	34 (69.4%)		197	154 (60.4%)	43 (67.2%)		197	82 (57.7%)	115 (65.0%)	
Pretreatment-BMI												
>18.5	282	254 (94.1%)	28 (57.1%)	**<0.001**	—	—	—		—	—	—	
≤18.5	37	16 (5.9%)	21 (42.9%)		—	—	—		—	—	—	
Preoperative-BMI												
>18.5	—	—	—		283	248 (97.3%)	35 (54.7%)	**<0.001**	—	—	—	
≤18.5	—	—	—		36	7 (2.7%)	29 (45.3%)		—	—	—	
Postoperative-BMI												
>18.5	—	—	—		—	—	—		283	137 (96.5%)	146 (82.5%)	**<0.001**
≤18.5	—	—	—		—	—	—		36	5 (3.5%)	31 (17.5%)	
Smoke												
No	153	133 (49.3%)	20 (40.8%)	0.276	153	131 (51.4%)	22 (34.4%)	**0.015**	153	77 (54.2%)	76 (42.9%)	**0.045**
Yes	166	137 (50.7%)	29 (59.2%)		166	124 (48.6%)	42 (65.6%)		166	65 (45.8%)	101 (57.1%)	
Coronary artery disease												
No	304	259 (95.9%)	45 (91.8%)	0.261	304	244 (95.7%)	60 (93.8%)	0.512	304	138 (97.2%)	166 (93.8%)	0.154
Yes	15	11 (4.1%)	4 (8.2%)		15	11 (4.3%)	4 (6.3%)		15	4 (2.8%)	11 (6.2%)	
Hypertension												
No	256	211 (78.1%)	45 (91.8%)	**0.027**	256	202 (79.2%)	54 (84.4%)	0.354	256	112 (78.9%)	144 (81.4%)	0.58
Yes	63	59 (21.9%)	4 (8.2%)		63	53 (20.8%)	10 (15.6%)		63	30 (21.1%)	33 (18.6%)	
COPD												
No	299	255 (94.4%)	44 (89.8%)	0.208	299	242 (94.9%)	57 (89.1%)	0.143	299	133 (93.7%)	166 (93.8%)	0.964
Yes	20	15 (5.6%)	5 (10.2%)		20	13 (5.1%)	7 (10.9%)		20	9 (6.3%)	11 (6.2%)	
Tumor location												
Upper third	48	42 (15.6%)	6 (12.2%)	0.745	48	39 (15.3%)	9 (14.1%)	0.636	48	23 (16.2%)	25 (14.1%)	0.837
Middle third	191	162 (60.0%)	29 (59.2%)		191	155 (60.8%)	36 (56.3%)		191	85 (59.9%)	106 (59.9%)	
Lower third	80	66 (24.4%)	14 (28.6%)		80	61 (23.9%)	19 (29.7%)		80	34 (23.9%)	46 (26.0%)	
Tumor diameter												
<=3.0	217	187 (69.3%)	30 (61.2%)	0.267	217	184 (72.2%)	33 (51.6%)	**0.002**	217	93 (65.5%)	124 (70.1%)	0.385
>3	102	83 (30.7%)	19 (38.8%)		102	71 (27.8%)	31 (48.4%)		102	49 (34.5%)	53 (29.9%)	
Pathological T stage												
pT0,1	170	151 (55.9%)	19 (38.8%)	**0.027**	170	143 (56.1%)	27 (42.2%)	**0.035**	170	85 (59.9%)	85 (48.0%)	**0.046**
pT2,3,4	149	119 (44.1%)	30 (61.2%)		149	112 (43.9%)	37 (57.8%)		149	57 (40.1%)	92 (52.0%)	
Pathological N stage												
pN negative	200	174 (64.4%)	26 (53.1%)	0.130	200	173 (67.8%)	27 (42.2%)	**<0.001**	200	98 (69.0%)	102 (57.6%)	**0.037**

pN positive	119	96 (35.6%)	23 (46.9%)		119	82 (32.2%)	37 (57.8%)		119	44 (31.0%)	75 (42.4%)	
Differentiation												
Well differentiated	147	128 (47.4%)	19 (38.8%)	0.408	147	122 (47.8%)	25 (39.1%)	0.433	147	76 (53.5%)	71 (40.1%)	**0.025**
Molecularly differentiated	76	61 (22.6%)	15 (30.6%)		76	58 (22.7%)	18 (28.1%)		76	25 (17.6%)	51 (28.8%)	
Poorly differentiated	96	81 (30.0%)	15 (30.6%)		96	75 (29.4%)	21 (32.8%)		96	41 (28.9%)	55 (31.1%)	
Preoperative treatment												
nCRT	293	246 (91.1%)	47 (95.9%)	0.395	293	234 (91.8%)	59 (92.2%)	0.912	293	130 (91.5%)	163 (92.1%)	0.861
nCT	26	24 (8.9%)	2 (4.1%)		26	21 (8.2%)	5 (7.8%)		26	12 (8.5%)	14 (7.9%)	
TRG												
TRG = 0,1	168	147 (54.4%)	21 (42.9%)	0.135	168	143 (56.1%)	25 (39.1%)	**0.015**	168	86 (60.6%)	82 (46.3%)	**0.011**
TRG = 2,3	151	123 (45.6%)	28 (57.1%)		151	112 (43.9%)	39 (60.9%)		151	56 (39.4%)	95 (53.7%)	

The bold *p*-values mean the *p* < 0.05. NRI, nutritional risk index; BMI, body mass index; CPOD, chronic obstructive pulmonary disease; ESCC, esophageal squamous cell carcinoma; nCRT, neoadjuvant chemoradiotherapy; nCT, neoadjuvant chemotherapy; TRG, tumor regression grade.

### 3.2 NRI and postoperative complications

Table 2 shows various factors such as preoperative chronic disease, sex, age, and patients’ nutritional status, evaluating indices such as BMI and NRI at different time points, and logistic regression analysis was used to explore the risk variables for postoperative complications. According to the logistic analysis, the univariate analysis results showed that complications after esophagectomy were more likely to occur in patients who underwent neoadjuvant chemotherapy (OR = 2.330, 95% CI: 1.038–5.228, *p* = 0.040) and those with low preoperative NRI (OR = 2.287, 95% CI: 1.303–4.012, *p* = 0.004). However, for patients with pretreatment NRI < 97.5, no significant association was found with higher postoperative complication rates, and the univariate analysis also demonstrated that BMI was not correlated with postoperative complications. Furthermore, the results of multivariate logistic regression also showed that preoperative NRI was an independent predictor of complications after esophagectomy (OR = 2.324, 95% CI 1.318–4.095, *p* = 0.004), but pretreatment NRI was not a risk factor for postoperative complications.

**Table 2:** Logistic regression analysis for clinical factors associated with complications after surgery.

Factors	Univariate analyses		Multivariate analyses	
	OR (95% CI)	*p*-value	OR (95% CI)	*p*-value
Sex (male/female)	1.285 (0.694–2.379)	0.424		
Age (≥60/<60)	1.252 (0.766–2.047)	0.369		
Smoke (yes/no)	1.457 (0.905–2.347)	0.121		
Coronary artery disease (present/absent)	0.776 (0.241–2.499)	0.671		
Hypertension (present/absent)	0.916 (0.504–1.667)	0.775		
COPD (present/absent)	2.286 (0.920–5.681)	0.075		
Preoperative treatment (nCT/nCRT)	2.330 (1.038–5.228)	**0.040**	2.406 (1.059–5.462)	**0.036**
Pretreatment BMI (low/high)	1.766 (0.878–3.550)	0.111		
Preoperative BMI (low/high)	1.434 (0.701–2.934)	0.324		
Pretreatment NRI (low/high)	1.308 (0.693–2.470)	0.408		
Preoperative NRI (low/high)	2.287 (1.303–4.012)	**0.004**	2.324 (1.318–4.095)	**0.004**
TRG (TRG = 2,3/ TRG0,1)	0.933 (0.582–1.496)	0.774		

The bold *p*-values mean the *p* < 0.05. NRI, nutritional risk index; BMI, body mass index; COPD, chronic obstructive pulmonary disease; nCRT, neoadjuvant chemoradiotherapy; nCT, neoadjuvant chemotherapy; TRG, tumor regression grade; OR, odds ratio; 95% CI, 95% confidence interval.

### 3.3 NRI and survival outcomes of esophagectomy following ESCC neoadjuvant therapy

**Figure 1:**
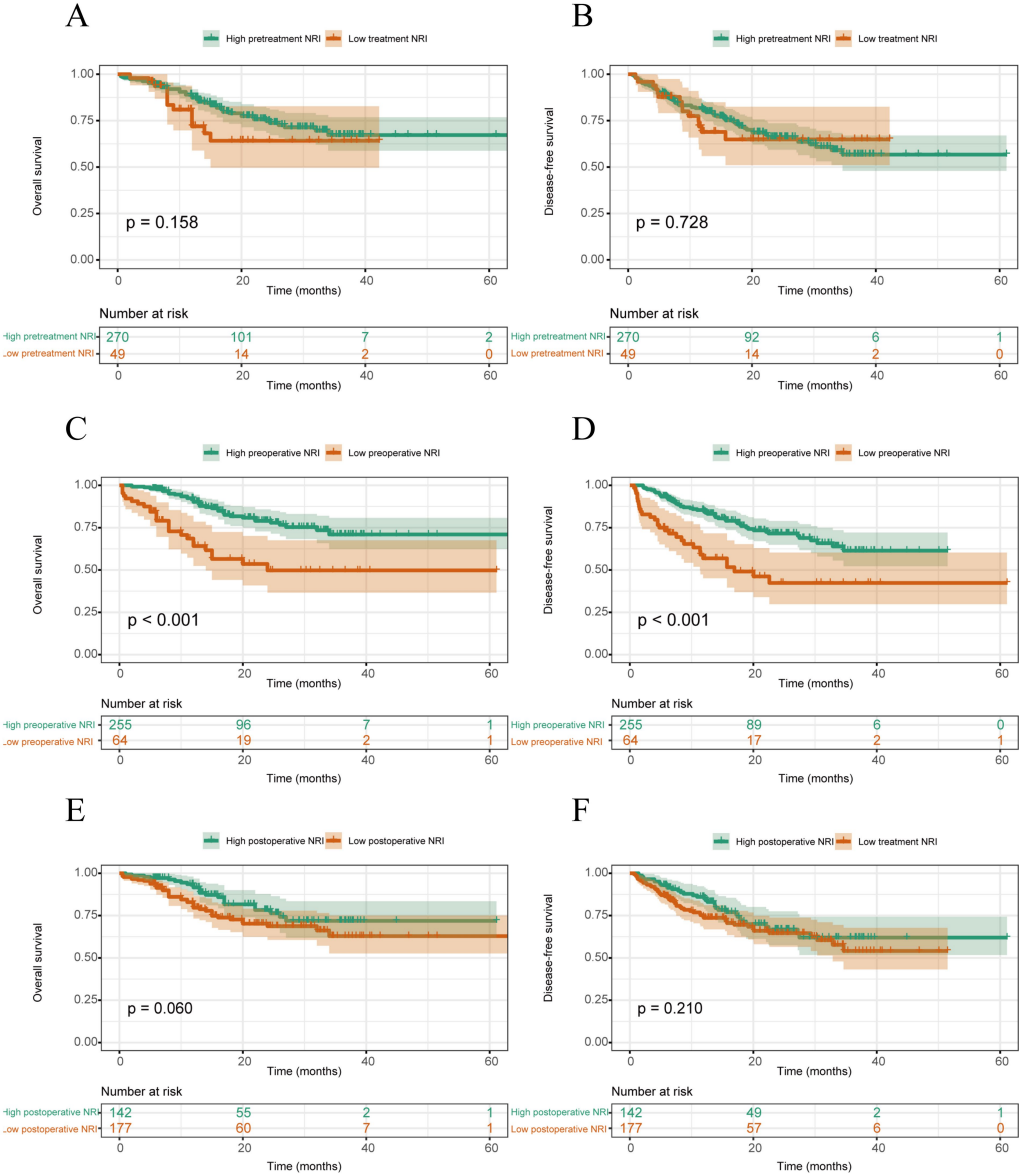
Survival curves stratified by pretreatment NRI: **(A)** overall survival and **(B)** disease-free survival of all study patients; survival curves stratified by preoperative NRI; **(C)** overall survival and **(D)** disease-free survival of all study patients; survival curves stratified by postoperative NRI; and **(E)** overall survival and **(F)** disease-free survival of all study patients.

The Kaplan–Meier curves for OS and DFS are shown in Figure 1, according to the cutoff value of the NRI. Based on the cutoff value of NRI set at 97.5, for patients with lower preoperative NRI, the OS (*p* < 0.001) and DFS (*p* < 0.001) were significantly decreased compared to those patients with preoperative NRI > 97.5 (Figure 1).

Univariate analysis revealed that sex (*p* = 0.021), smoking (*p* = 0.044), tumor size (*p* = 0.002), pT stage (*p* = 0.001), pN stage (*p* < 0.001), tumor differentiation (*p* = 0.001), tumor regression grade (TRG) after neoadjuvant therapy (*p* < 0.001), pretreatment BMI (*p* = 0.014), postoperative BMI (*p* = 0.030), and preoperative NRI (*p* < 0.001) were prognostic factors significantly affecting the OS of ESCC patients. Meanwhile, based on the multivariate analysis, the preoperative NRI (HR, 2.005; 95% CI, 1.070–3.760; *p* = 0 0.030) and pN stage (HR, 2.769; 95% CI, 1.549–4.948; *p* = 0.001) were also independent prognostic factors for OS (Table 3).

**Table 3:** Uni- and multivariate analyses for the overall survival of 319 patients with ESCC.

Factors	Univariate analyses		Multivariate analyses	
	HR (95% CI)	*p*-value	HR (95% CI)	*p*-value
Sex (male/female)	2.700 (1.165–6.256)	**0.021**	1.539 (0.588–4.025)	0.380
Age (≥60/<60)	1.070 (0.649–1.764)	0.791		
Smoke (yes/no)	1.838 (1.092–3.091)	**0.044**	1.381 (0.751–2.540)	0.299
Coronary artery disease (present/absent)	2.193 (0.793–6.062)	0.130		
Hypertension (present/absent)	1.000 (0.534–1.872)	1.000		
COPD (present/absent)	0.411 (0.057–2.977)	0.379		
Tumor location	1.068 (0.708–1.610)	0.755		
Tumor diameter (≥3/<3 cm)	2.130 (1.308–3.470)	**0.002**	1.383 (0.809–2.364)	0.236
pT (2,3,4/0,1)	2.361 (1.412–3.947)	**0.001**	0.833 (0.341–2.040)	0.690
pN (positive/negative)	4.082 (2.436–6.839)	**<0.001**	2.769 (1.549–4.948)	**0.001**
Differentiation	1.643 (1.236–2.183)	**0.001**	1.239 (0.763–2.011)	0.386
TRG (TRG = 2,3/TRG0,1)	2.589 (1.538–4.356)	**<0.001**	1.166 (0.496–2.738)	0.725
Preoperative treatment (nCT/nCRT)	0.811 (0.195–3.365)	0.772		
Pretreatment BMI (low/high)	2.151 (1.170–3.954)	**0.014**	1.551 (0.396–6.078)	0.529
Preoperative BMI (low/high)	1.751 (0.915–3.350)	0.091		
Postoperative BMI (low/high)	2.002 (1.069–3.750)	**0.030**	0.652 (0.165–2.570)	0.541
Pretreatment NRI (low/high)	1.542 (0.839–2.836)	0.163		
Preoperative NRI (low/high)	3.139 (1.903–5.177)	**<0.001**	2.005 (1.070–3.760)	**0.030**
Postoperative NRI (low/high)	1.618 (0.973–2.692)	0.064		

The bold *p*-values mean the *p* < 0.05. NRI, nutritional risk index; BMI, body mass index; COPD, chronic obstructive pulmonary disease; ESCC, esophageal squamous cell cancer; nCRT, neoadjuvant chemoradiotherapy; nCT, neoadjuvant chemotherapy; HR, hazard ratio; 95% CI, 95% confidence interval; TRG, tumor regression grade.

Regarding the DFS of ESCC patients (Table 4), univariate analysis revealed that sex (*p* = 0.045), smoking (*p* = 0.006), tumor size (*p* = 0.001), pT stage (*p* < 0.001), pN stage (*p* < 0.001), tumor differentiation (*p* < 0.001), TRG (<0.001), and preoperative NRI (*p* < 0.001) were prognostic factors significantly associated with the DFS of ESCC patients, while preoperative NRI (HR, 1.736; 95% CI, 1.086–2.775; *p* = 0.021) and pN stage (HR, 2.535; 95% CI, 1.566–4.104; *p* < 0.001) were independent prognostic factors, in accordance with previous results.

**Table 4:** Uni- and multivariate analyses for the disease-free survival of 319 EC patients.

Factors	Univariate analyses		Multivariate analyses	
	HR (95% CI)	*p* value	HR (95% CI)	*p*-value
Sex (male/female)	1.910 (1.015–3.594)	**0.045**	0.995 (0.466–2.126)	0.990
Age (≥60/<60)	1.050 (0.683–1.614)	0.824		
Smoke (yes/no)	1.860 (1.192–2.902)	**0.006**	1.671 (0.974–2.866)	0.062
Coronary artery disease (present/absent)	1.741 (0.703–4.313)	0.231		
Hypertension (present/absent)	0.988 (0.574–1.699)	0.965		
COPD (present/absent)	1.047 (0.382–2.868)	0.929		
Tumor location	1.088 (0.768–1.541)	0.634		
Tumor diameter (≥3/<3 cm)	2.027 (1.333–3.080)	**0.001**	1.371 (0.871–2.161)	0.173
pT (2,3,4/0,1)	2.349 (1.516–3.638)	**<0.001**	1.139 (0.551–2.352)	0.726
pN (positive/negative)	3.416 (2.219–5.259)	**<0.001**	2.535 (1.566–4.104)	**<0.001**
Differentiation	1.579 (1.238–2.014)	**<0.001**	1.105 (0.745–1.641)	0.620
TRG (TRG = 2,3/TRG0,1)	2.380 (1.536–3.688)	**<0.001**	1.099 (0.543–2.221)	0.794
Preoperative treatment (nCT/nCRT)	0.469 (0.114–1.921)	0.292		
Pretreatment BMI (low/high)	1.642 (0.927–2.909)	0.089		
Preoperative BMI (low/high)	1.496 (0.830–2.697)	0.180		
Postoperative BMI (low/high)	1.692 (0.955–2.997)	0.072		
Pretreatment NRI (low/high)	1.107 (0.625–1.960)	0.728		
Preoperative NRI (low/high)	2.609 (1.678–4.056)	**<0.001**	1.736 (1.086–2.775)	**0.021**
Postoperative NRI (low/high)	1.313 (0.857–2.013)	0.211		

The bold *p*-values mean the *p* < 0.05. NRI, nutritional risk index; BMI, body mass index; COPD, chronic obstructive pulmonary disease; ESCC, esophageal squamous cell cancer; nCRT, neoadjuvant chemoradiotherapy; nCT, neoadjuvant chemotherapy; HR, hazard ratio; 95% CI: 95% confidence interval; TRG, tumor regression grade.

### 3.4 Subgroup analysis

**Figure 2:**
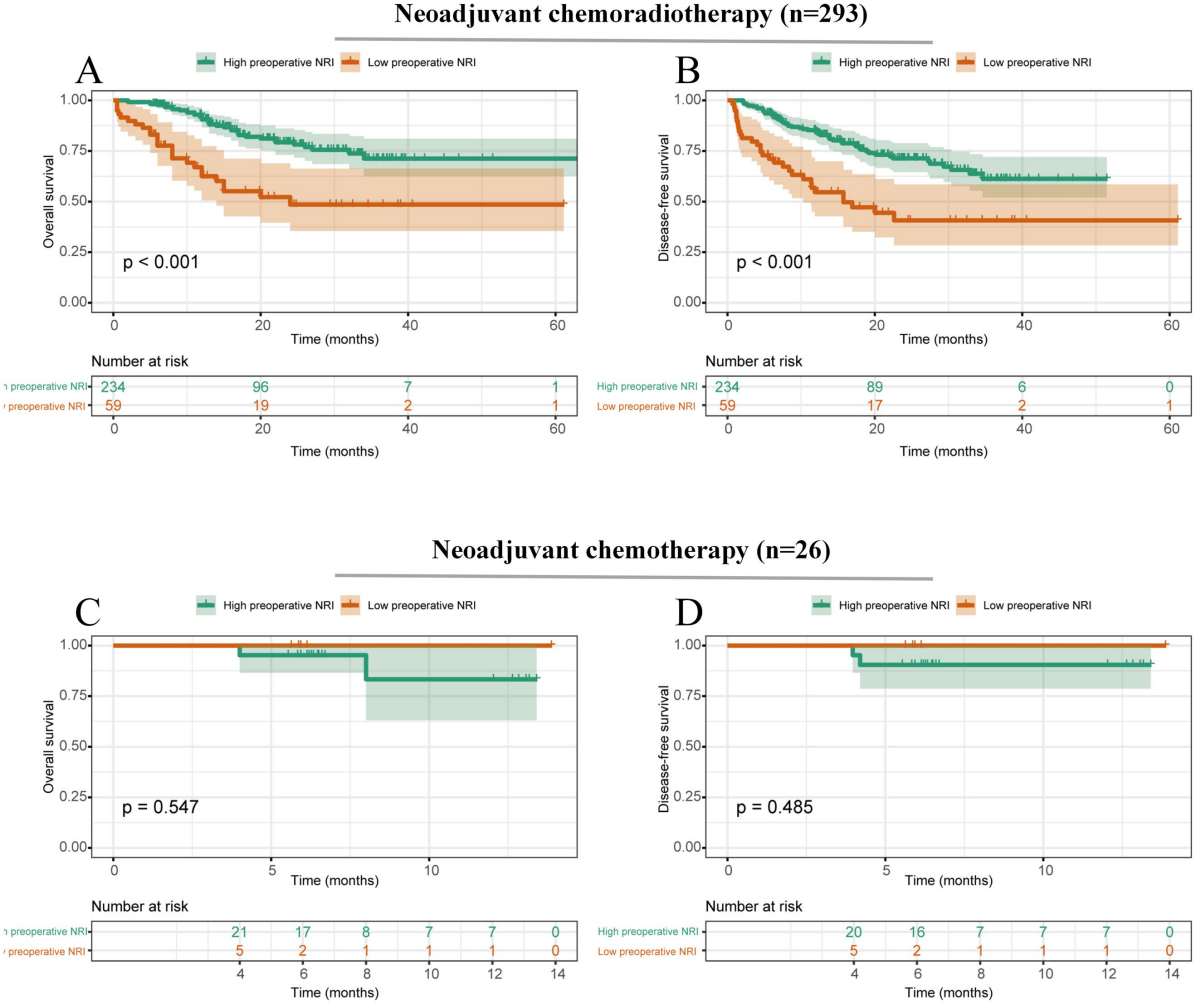
Survival curves stratified by preoperative NRI based on different preoperative treatments. **(A)** Overall survival of patients with ESCC who underwent neoadjuvant chemoradiotherapy; **(B)** Disease-free survival of patients with ESCC who underwent neoadjuvant chemoradiotherapy; **(C)** Overall survival of patients with ESCC who underwent neoadjuvant chemotherapy; and **(D)** Disease-free survival of patients with ESCC who underwent neoadjuvant chemotherapy.

We conducted a subgroup analysis to detect the impact of preoperative NRI on the survival outcomes of ESCC patients who received different preoperative therapies. The results showed that a low preoperative NRI was associated with poorer OS (*p* < 0.001) and DFS (*p* < 0.001) in patients who had undergone neoadjuvant chemoradiotherapy (*n* = 293). However, this correlation was not detected in patients treated with neoadjuvant chemotherapy (*n* = 26) (Figure 2).

**Figure 3:**
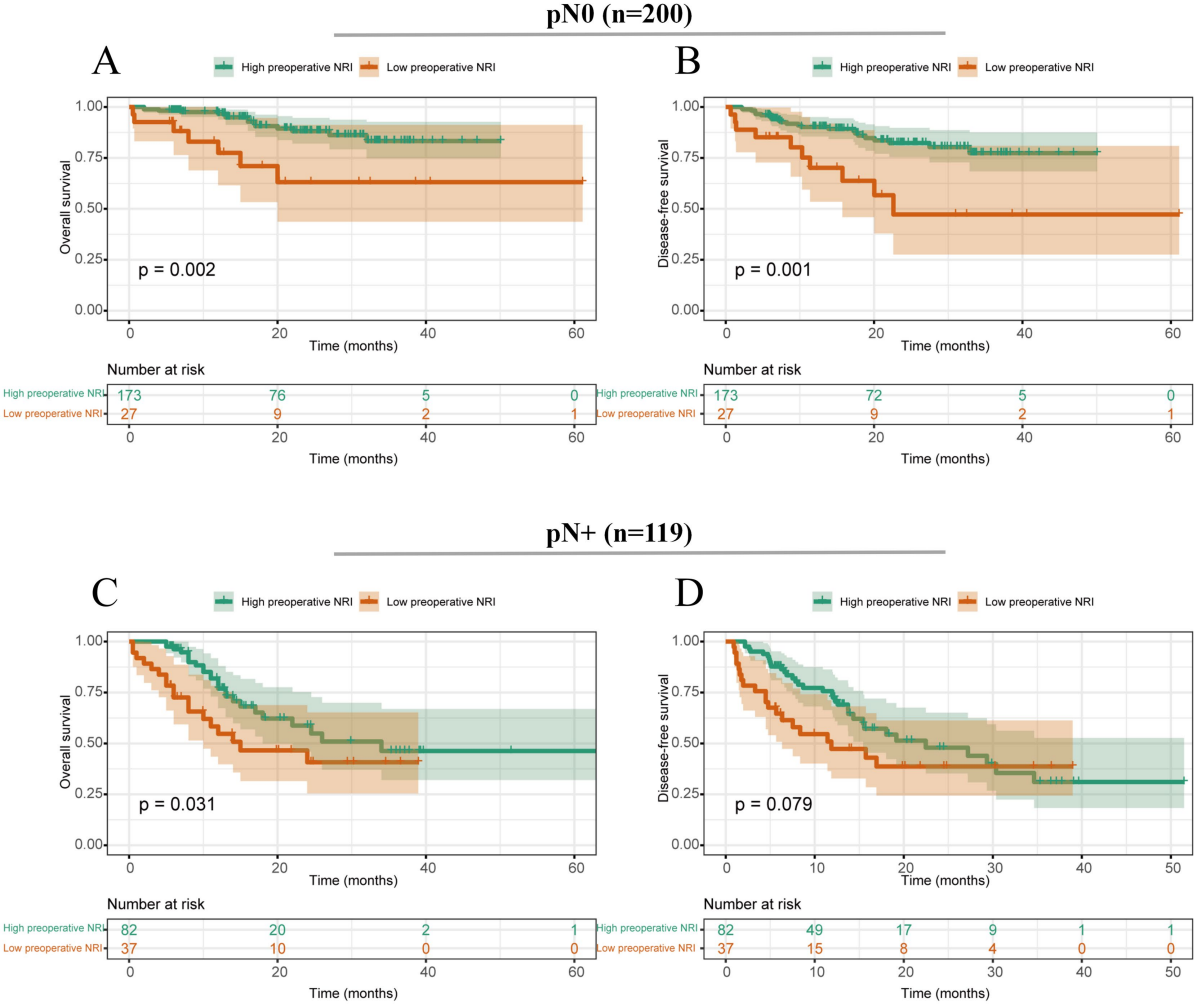
Survival curves stratified by preoperative NRI based on different tumor stages. **(A)** Overall survival of patients with ESCC with negative N stage; **(B)** Disease-free survival of patients with ESCC with negative N stage; **(C)** Overall survival of patients with ESCC with positive N stage; **(D)** Disease-free survival of patients with ESCC with positive N stage.

Moreover, we conducted a subgroup analysis to detect the impact of preoperative NRI on survival outcomes of ESCC patients with (*n* = 200) or without lymph node metastasis (*n* = 119). The subgroup analysis showed that a lower preoperative NRI was significantly associated with poor OS in both N-negative (*p* = 0.002) and N-positive (*p* = 0.031) ESCC patients (Figure 3).

## 4 Discussion

In ESCC patients, various nutritional indices such as BMI and serum albumin levels were proven to be correlated with cancer progression and prognosis; some of them have already been widely used in clinics to evaluate the nutritional level of patients (22–25). As a new index in nutritional evaluation, the NRI is a simple, objective nutritional evaluation score that is calculated using weight, height, and albumin in peripheral blood and is easily obtained from routine blood examinations. Many scholars have applied NRI not only as a nutritional evaluation index but also as a prognostic factor in malignancies. Justin et al. found that in head and neck cancer patients who were treated with chemo-radiotherapy, NRI could predict the OS and be associated with composite complications (26). Kim et al. showed that preoperative NRI is a predictor of OS in gastric cancer patients; patients with high NRI had a longer survival compared with patients with low NRI, and NRI could be used as an indicator for accurate prediction of the prognosis in patients with gastric cancer (9). However, the clinical implications of ESCC remain unclear. Therefore, our study aimed to investigate the prognostic value of the NRI in ESCC.

Our study is the first to retrospectively utilize NRI to assess prognosis in a relatively large group of ESCC patients. In this study, we assessed the correlation between NRI at different treatment time points and compared NRI with the traditional nutritional index BMI to evaluate the risk of postoperative complications, clinicopathological factors, and survival outcomes of ESCC patients who had been treated with neoadjuvant therapy followed by esophagectomy in a single, high-volume institute. The results of this study revealed that ESCC patients with low preoperative NRI were associated with poor survival outcomes in the univariate analysis. In addition, multivariate analysis showed that preoperative NRI was an independent prognostic factor for OS and DFS, and preoperative NRI was also proven to be an independent risk factor for postoperative complications. Furthermore, pretreatment NRI has been proven to have no significant impact on OS (*p* = 0.163) or DFS (*p* = 0.728). Univariate analysis also indicated that postoperative NRI was not correlated with OS (*p* = 0.064) or DFS (*p* = 0.211). It is suggested that for postoperative complications and survival outcomes in ESCC patients followed by neoadjuvant therapy, preoperative NRI might be a superior index compared with NRI at other time points.

Additionally, the classical nutritional evaluation parameter BMI was proved not to be independently associated with the survival outcomes and postoperative complications based on the results of multivariate analysis, which may suggest that preoperative NRI was superior to BMI in predicting the prognosis of ESCC patients. This study was in accordance with previous studies that detected the prognostic role of NRI in other malignancies (27–29).

The superior prognostic value of the preoperative NRI likely reflects the integration of both acute and cumulative nutritional and systemic responses to neoadjuvant therapy. Neoadjuvant chemoradiotherapy induces profound metabolic and immunological changes, including inflammation-driven catabolism, anorexia, mucosal injury, and altered protein synthesis (30). These systemic effects often culminate in a decline in serum albumin and body weight—both key components of the NRI. As such, the preoperative NRI reflects a patient’s nutritional and inflammatory status after completing neoadjuvant therapy but prior to surgical intervention, thereby capturing both treatment-induced physiological stress and residual host reserve. This makes it particularly useful for predicting postoperative complications and long-term survival outcomes. In contrast, pretreatment NRI merely reflects the baseline nutritional status before therapy-induced physiological stress begins and thus may underestimate the risk in patients who later experience treatment-related nutritional deterioration. Meanwhile, postoperative NRI may be influenced by surgical stress, fluid shifts, and acute-phase responses, which can transiently alter albumin and weight independent of cancer-related cachexia or baseline reserves (31). We further agree that it is essential to compare the NRI with BMI. While BMI is a commonly used anthropometric index that reflects general body mass, it lacks sensitivity to protein reserves or inflammatory states and cannot distinguish between lean tissue and fat mass. In contrast, NRI incorporates serum albumin, a surrogate marker of hepatic protein synthesis and systemic inflammation, thereby offering a more dynamic and integrative assessment of nutritional and immune status (32). Despite its statistical non-significance in our multivariate model, BMI still provided complementary value by reflecting long-term nutritional trends, particularly in identifying underweight individuals with potential baseline vulnerability. Therefore, we believe that NRI and BMI may serve synergistic roles: BMI as a structural measure and NRI as a functional indicator of the nutritional and inflammatory status.

The results of the subgroup analysis indicated that preoperative NRI was associated with poor survival in ESCC patients who underwent neoadjuvant chemoradiotherapy but not in the neoadjuvant chemotherapy group. This discrepancy might be partly explained by the insufficient sample size of only 26 ESCC patients who underwent neoadjuvant chemotherapy. Therefore, clinical research with a larger sample size is necessary to elucidate the true results. Subgroup analysis results also demonstrated that whether patients had or did not have lymph node metastasis, the preoperative NRI was still proven to be associated with survival outcomes in patients with ESCC. Therefore, these findings highlight the importance of considering preoperative nutritional status as a potential prognostic factor in ESCC patients and emphasize the need for comprehensive assessment and management of nutritional status during the preoperative period.

Considering the robust predictive value of preoperative NRI in ESCC patients, the NRI might have the potential to provide nutritional support during the preoperative management of ESCC patients. Liu et al. (33) had proved that in patients with EC, preoperative nutritional and home enteral nutritional support is feasible, safe, and beneficial to EC patients treated by esophagectomy. Przekop et al. (34) also suggested that nutritional support should be implemented in the early treatment period of tumors and patients would gain more benefits. Therefore, combining the preoperative NRI results and nutritional management in ESCC patients seems practical. It is indicated that in ESCC patients with a low NRI, providing adequate nutritional support might reduce postoperative complications and ameliorate the patient survival.

Despite the strengths of our study, several limitations should be acknowledged. First, this was a single-center retrospective study, which may have introduced inherent biases, including variability in clinical management and non-standardized nutritional interventions before surgery. Second, although we adopted a widely referenced NRI cutoff value of 97.5, this threshold may not be generalizable across different populations or clinical settings due to ethnic, dietary, and healthcare differences. Future multicenter studies are needed to validate and calibrate the optimal NRI cutoff values. Third, our analysis did not include direct comparisons with other established nutritional indices, such as the Controlling Nutritional Status (CONUT) score or the Patient-Generated Subjective Global Assessment (PG-SGA), which may provide complementary or superior prognostic value. Incorporating these indices in future prospective studies may help determine the most effective tool for nutritional risk stratification in ESCC patients.

## 5 Conclusion

This study suggests that preoperative NRI can be used in the evaluation of nutritional status and that preoperative NRI, rather than pretreatment or postoperative NRI, is an independent prognostic factor of postoperative complications and survival in ESCC patients who underwent neoadjuvant therapy followed by surgery.

## Data Availability

The raw data supporting the conclusions of this article will be made available by the authors, without undue reservation.
