# Supplementary figures and images for: Preoperative NRI outperforms other time points in predicting prognosis of ESCC with neoadjuvant therapy
Source: Front Nutr. 2025 Jun 25;12:1613868. doi: 10.3389/fnut.2025.1613868 (PMC12237673; doi:10.3389/fnut.2025.1613868)

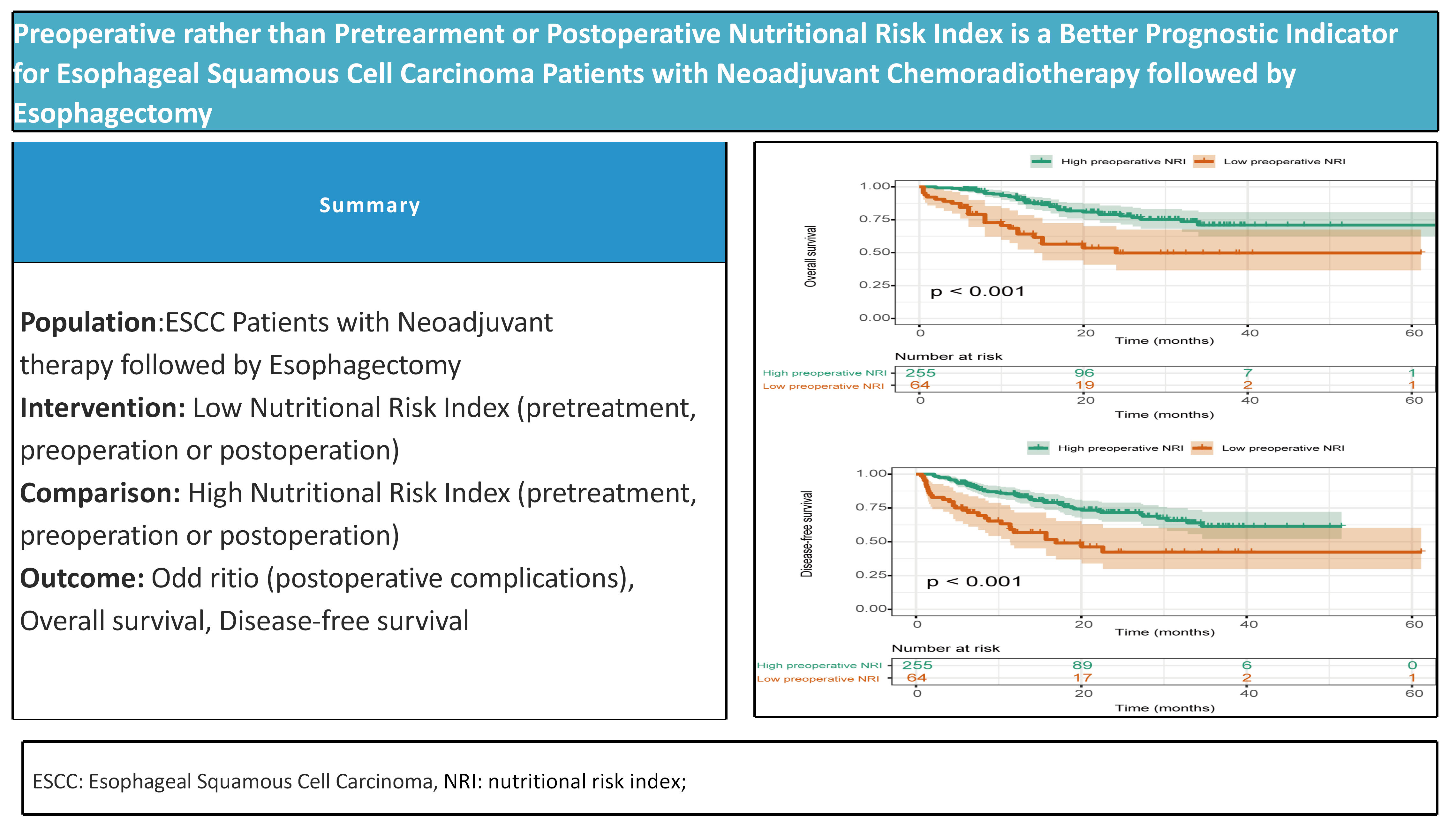

Supplement: Supplementary file 1 [file Image_1.tif]
